# Effect of Ultrasonic Treatment on Structure and Physicochemical Properties of Pea Starch

**DOI:** 10.3390/foods12132620

**Published:** 2023-07-06

**Authors:** Gang Li, Xiaohong Ge, Changsheng Guo, Benguo Liu

**Affiliations:** School of Food Science, Henan Institute of Science and Technology, Xinxiang 453003, China; ligang@hist.edu.cn (G.L.); gexh@hist.edu.cn (X.G.); guo1186958122@163.com (C.G.)

**Keywords:** pea starch, ultrasonic modification, physicochemical properties, characterization

## Abstract

The effects of ultrasonic treatment on the structure and physicochemical properties of pea starch were investigated in this study. The results showed that ultrasonic treatment increased the hydrolysis rate and particle size of pea starch. In the process of treatment, there were some depressions and pores on the surface of pea starch granules. Although the crystallization type of starch was retained, its crystallinity decreased. The pasting temperature of pea starch remained stable after ultrasonic treatment, but its peak viscosity, trough viscosity, cold viscosity, breakdown viscosity and setback viscosity all declined significantly. The transparency of starch paste decreased, but proper ultrasonic treatment could improve the strength of starch gel. The obtained results can provide a reference for the physical modification of pea starch.

## 1. Introduction

As the second largest edible legume in the world, the pea is rich in nutrients such as starch and protein, and its starch content is about 45% to 55%. Compared with corn starch and potato starch, pea starch is easy to extract and cheap [1,2]. However, due to its poor physicochemical properties, natural pea starch has some shortcomings, such as weak shear resistance, poor acid resistance, low thermal stability, high sedimentation and so on. Its properties need to be changed via physical, chemical or enzyme modification methods to expand its application scope [3,4]. With the development of green processing, research on the physical modification of starch is increasing rapidly. At present, the methods of the physical modification of starch include extrusion, microwave, radiation, ultrasound and so on [5]. Ultrasonic modification has attracted the wide attention of researchers as a physical modification method with safety, environmental protection, high efficiency and low energy consumption [6].

Ultrasonic treatment mainly promotes the movement of starch molecular chains through mechanical effect, cavitation effect and thermal effect, changes the original hydrogen bond and double helix structure in starch granules and regulates the physicochemical properties of starch through the influence on the surface and internal structure of starch granules [7,8]. Hu et al. used dual-frequency (20 kHz + 25 kHz) ultrasound to treat corn starch and obtained modified starch with significantly improved thermal stability and regeneration [9]. Karwasra et al. found that the ultrasonic treatment could enhance the oil absorption capacity, amylose content and swelling power of Indian wheat cultivar starches [10].

In this study, the effects of ultrasonic treatment time on the structure and physicochemical properties of pea starch were systematically investigated, in order to provide a theoretical basis for the development of modified pea starch.

## 2. Materials and Methods

### 2.1. Materials and Chemicals

Pea starch, glucose, phenol and sulfuric acid were purchased from Shanghai Yuanye Biotechnology Co., Ltd. (Shanghai, China). Ultra-pure water was produced using a Thermo Genpure UV/UF water system (Waltham, MA, USA).

### 2.2. Ultrasonic Treatment of Pea Starch

Starch was suspended in 100 mL distilled water with a concentration of 20% (*w*/*w*) and loaded on a SCIENTZ JY99-IIDN ultrasonic homogenizer (Ningbo, China). The sample vessel was cooled using an ice bath. The sample was treated at 680 W for 10, 20 and 30 min. The action time and interval time of ultrasound were set to 2 s. After ultrasonic treatment, the starch was washed with distilled water and dried at 40 °C.

### 2.3. Determination of Hydrolysis Rate

The treated starch was diluted properly with water. One milliliter sample solution was put into a test tube, shaken with 5% phenol solution and 3 mL sulfuric acid, kept at 30 °C for 15 min and cooled to room temperature. Its absorbance was determined at 490 nm through the use of a Persee TU-1810 spectrophotometer (Beijing, China). By comparing it with the standard curve of glucose, the amount of glucose produced via hydrolysis was determined and the hydrolysis rate was calculated [11].

### 2.4. Microscopic Observation Method

Native and treated pea starch were evenly coated on the conductive adhesive attached to the loading platform, and then sent to the sample compartment after vacuum gold spraying. An FEI Quanta 200 environmental scanning electron microscope (Hillsboro, OH, USA) was used to observe the starch particles under the magnification of 2000 times, and the representative pictures were recorded via microscope [12].

### 2.5. Polarizing Microscopic Observation Method

An appropriate amount of 1% starch solution was dropped on the slide, covered with the cover slide and placed on the loading platform. Polarizing characteristics of starch were observed through the use of a Hengping BH200P polarizing microscope (Shanghai, China) 200 times [13].

### 2.6. Measurement of Particle Size Distribution

After the appropriate dilution of starch solution, particle size distribution was determined using a BETTER BT-9300H laser particle analyzer (Dandong, China).

### 2.7. Determination of X-ray Diffraction Pattern

An appropriate amount of starch was taken into the sample tank, the excess sample was scraped off and the surface of the sample was kept smooth. A Rigaku Smartlab SE X-ray diffractometer (Tokyo, Japan) was used to determine the crystalline structure of starch. Test parameters: scanning rate, 8°/min; scanning range, 5–70°; step size, 0.02°. The crystallinity of the sample was calculated using JADE6.0 software (Materials Data Inc., Livermore, CA, USA).

### 2.8. Transparency Measurement

A total of 20 mL of 1% starch suspension was heated in a boiling water bath for 15 min. The light transmittance of starch paste at 620 nm after cooling was measured through the use of a Persee TU-1810 spectrophotometer (Beijing, China) with water as the control [14].

### 2.9. Evaluation of Pasting Properties

A 3.0 g starch sample was mixed with 25 mL distilled water and placed in a Perten RVA 4500 rapid visco analyzer (Stockholm, Sweden) [15]. The sample was kept at 50 °C for l min, heated up to 95 °C within 3.5 min and kept for 3 min and then dropped to 50 °C within 3.5 min and kept for 2 min. The viscosity change in the sample was recorded during this process.

### 2.10. Determination of Starch Gel Strength

The starch sample (3.2 g) was put into a 50 mL beaker and distilled water was added until the total mass was 40.0 g. The starch solution was heated at 95 °C and stirred for 30 min until the starch had gelatinized completely. The starch that had been cooled to room temperature was transferred to a refrigerator and kept at 4 °C for 16 h to form a stable starch gel sample. The strength of starch gel was determined using a TA-XT Plus texture analyzer (Stable Micro Systems, Surrey, UK) with a P/0.5 probe. The measurement parameters were set as follows: trigger force, 2 g; compression distance, 10 mm; descending speed, measuring speed and rising speed of the probe: 1.5, 1.0 and 1.0 mm/s, respectively [13].

### 2.11. Statistical Analysis

All experiments were repeated three times and expressed as mean ± standard deviation. The statistical comparison was based on Duncan’s test with a confidence level of 95%. Origin 8.0 software (OriginLab Corporation, Northampton, MA, USA) was used for statistical processing and plotting.

## 3. Results and Discussion

### 3.1. Hydrolysis Rate

**Figure 1:**
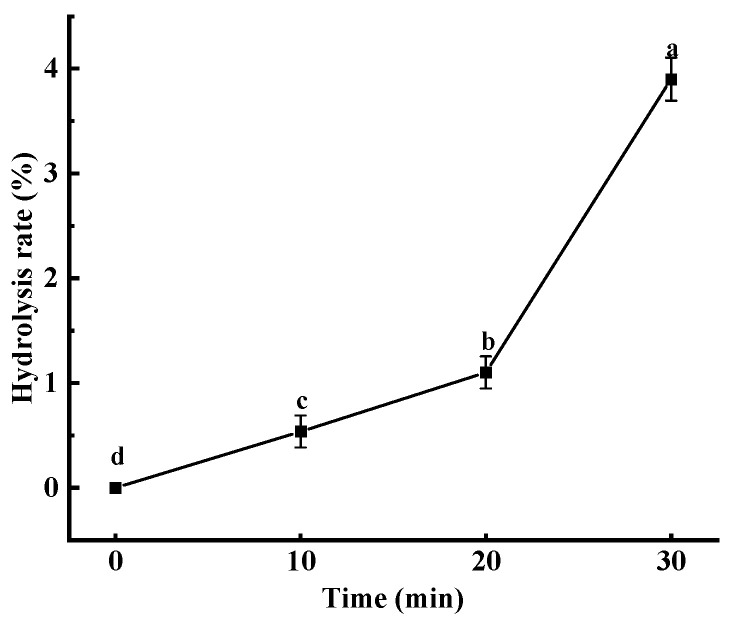
Effect of ultrasonic treatment time on the hydrolysis rate of pea starch. Different letters above the line indicate a significant difference (*p* < 0.05).

Studies on the modification of pea starch via heat–moisture treatment [16], amylomaltase [17] and octenyl succinic anhydride [18] have been reported, but there has not been a study on the modification of pea starch via ultrasonic treatment. In this study, the effects of ultrasonic treatment on the structure and physicochemical properties of pea starch were systematically investigated. The effect of ultrasonic treatment on the hydrolysis rate of pea starch is shown in Figure 1. With the extension of ultrasonic treatment time, the hydrolysis rate of starch increased gradually. The hydrolysis rate of starch increased from 0.5% at 10 min to 4% at 30 min. The ultrasound acted on the liquid medium to form mechanical vibrations, which in turn caused a thermal effect, mechanical effect and cavitation effect, leading to the fracture of the starch chains [19]. The longer the ultrasonic time, the higher the degree of damage; so, the hydrolysis rate of starch was increased.

### 3.2. SEM Analysis

**Figure 2:**
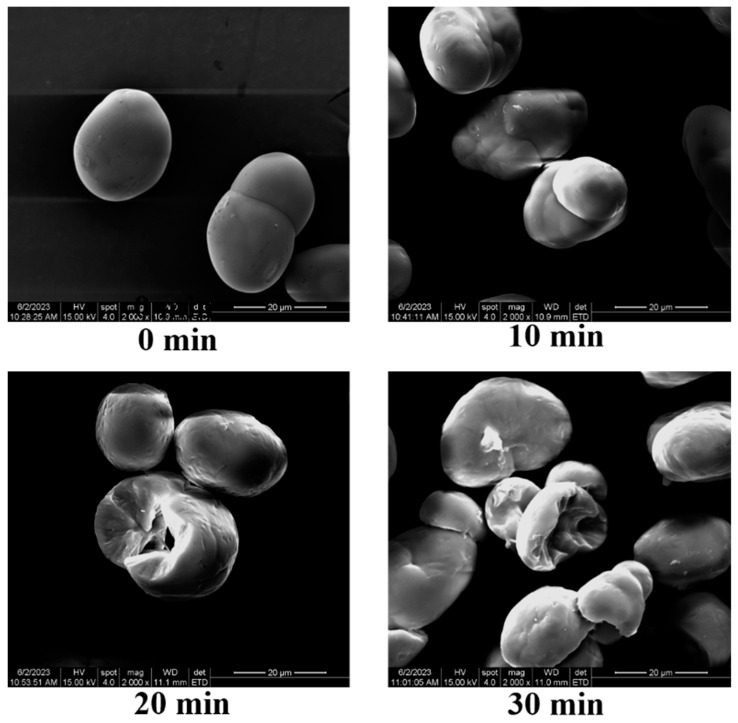
Effect of ultrasonic treatment time on the scanning electron microscope image of pea starch.

Ultrasonic treatment will change the morphology of starch particles, such as cracks, collapses, holes, etc., whereby the extent of which depends on the intensity of the ultrasonic treatment [20]. Figure 2 demonstrates the effect of ultrasonic treatment time on the particle morphology of pea starch. The native pea starch granules were oval, their surface and edges were relatively smooth and the granules were complete. The shape and size of the pea starch granules changed after ultrasonic treatment. The damage to the starch granules caused by ultrasonic treatment for a short time was relatively small, and a small amount of shallow damage appeared on the surface. With the extension of treatment time, especially within 30 min, the damage on the surface of the starch granules became more prominent, the edges, corners and surface became rough and some depressions and pores appeared. The main reason for this is that the high energy produced via ultrasonic cavitation and mechanical action accelerates the movement of starch particles, and the high shear force and high-frequency microjet act on the surface of starch particles, resulting in varying degrees of damage and destruction to the morphology of the starch granules.

### 3.3. Polarized Light Microscopic Analysis

**Figure 3:**
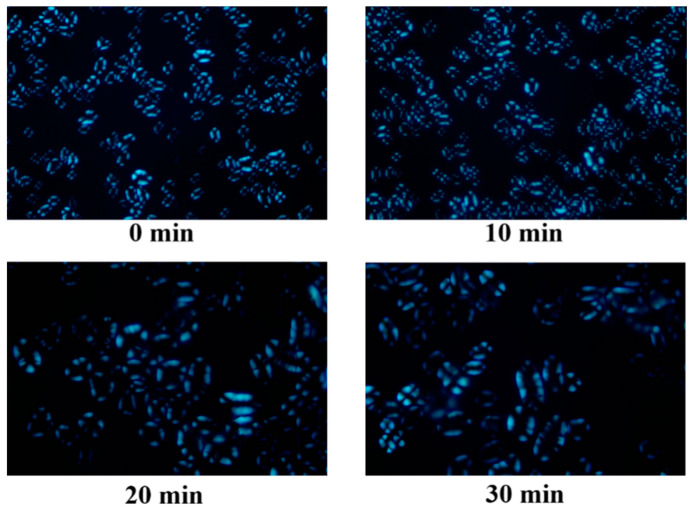
Effect of ultrasonic treatment time on the polarized light microscope image of pea starch.

The formation of a polarized cross originates from the spherulite structure of starch. When polarized light passes through starch particles, an obvious polarized cross is observed, which can reflect changes in the molecular chain arrangement and crystal structure of starch [21]. Figure 3 exhibits the polarizing microscope images of pea starch with different ultrasonic treatment times. An obvious polarization cross could be observed, which suggests that ultrasonic treatment did not damage the spherulite structure of starch, and the starch basically maintained its original molecular arrangement and structure. However, after the treatment of 30 min, the particles gathered.

### 3.4. Particle Size Analysis

Starch granules from different cereals have their own unique shapes and sizes, and the size of starch granules has an important influence on the properties of starch. There are differences in gel properties, gelatinization, retrogradation and enzymatic hydrolysis properties of starch particles with different sizes [22]. The effect of ultrasonic treatment time on the size of pea starch is shown in Table 1. The median particle size of untreated pea starch was 18.233 μm, exhibiting a unimodal distribution. After ultrasonic treatment, the particle size became larger. After ultrasonic treatment of 30 min, the median particle size of pea starch increased to 20.853 μm. This is mainly due to the fact that after ultrasonic treatment, the internal structure of starch particles becomes loose, pores and grooves appear and it is easier for them to absorb water and expand when dispersed in water; secondly, the surface of starch particles is destroyed and the surface activity is increased, which is more prone to agglomeration behavior, resulting in an increase in particle size.

### 3.5. XRD Analysis

**Figure 4:**
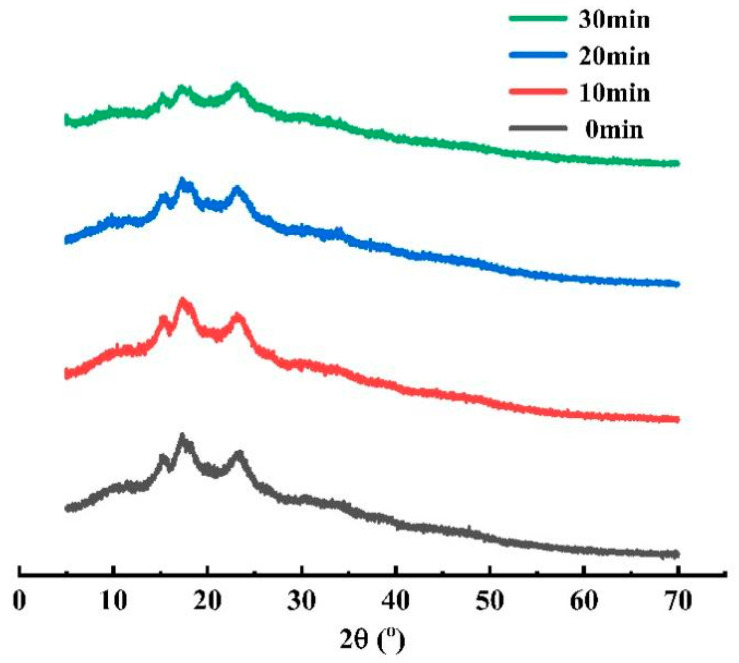
Effect of ultrasonic treatment time on the XRD pattern of pea starch.

The common XRD patterns of starch are A-type, B-type and C-type [23]. As shown in Figure 4, the native pea starch had strong diffraction peaks at 15.3°, 17.3°and 23.1°, which were in accordance with the characteristics of C-type crystallization. There was no significant difference in the peak position of the diffraction pattern of pea starch before and after ultrasound, indicating that ultrasound preferentially acted on the amorphous region and did not destroy the crystal form of pea starch. After ultrasonic treatment, the diffraction peak intensity of pea starch declined slightly, especially at 23°. However, the extension of ultrasonic time did not cause a significant change in the crystallinity of pea starch. The crystallinity of native pea starch was 26.3%, and that of pea starch after ultrasonic treatment of 30 min was 24.9%. Kaul et al. [24] also found a similar phenomenon in the study of ultrasonic modification of potato starch.

### 3.6. Transparency Analysis

**Figure 5:**
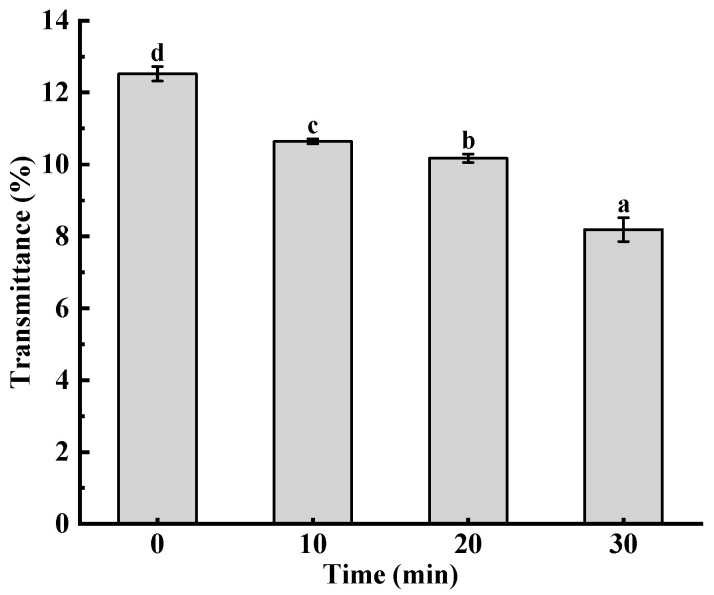
Effect of ultrasonic treatment time on the transparency of pea starch. Different letters above the bar indicate a significant difference (*p* < 0.05).

The transparency of starch paste is reflected by the transmittance of starch paste [25], and the effect of ultrasonic treatment on the transparency of pea starch is shown in Figure 5. With an increase in ultrasonic time, the transparency of the starch paste declined gradually. This might be because ultrasonic treatment made the surface of starch granules rougher after destruction, and it was easier for the granules to expand and absorb water, thus increasing solubility, but long-term treatment produced more short-amylose molecules, which were easier to gather, resulting in starch condensation. Therefore, it exhibited lower transparency.

### 3.7. Analysis of Pasting Properties

The gelatinization of starch is the thermal disorder of the crystal structure of starch granules, which is not only related to the type of starch, system temperature and pH, but is also affected by the crystal structure of starch and the ratio of amylose to amylopectin [26]. In Table 2, the peak viscosity, trough viscosity and cold viscosity of native pea starch are shown to be 3990, 3012 and 6052 cP, respectively. After ultrasonic treatment of 30 min, the corresponding viscosity values were 3582, 3070 and 5596 cP, respectively. There was no significant change in pasting temperature. Both breakdown viscosity and setback viscosity also decreased with the extension of treatment time.

### 3.8. Analysis of Gel Strength

**Figure 6:**
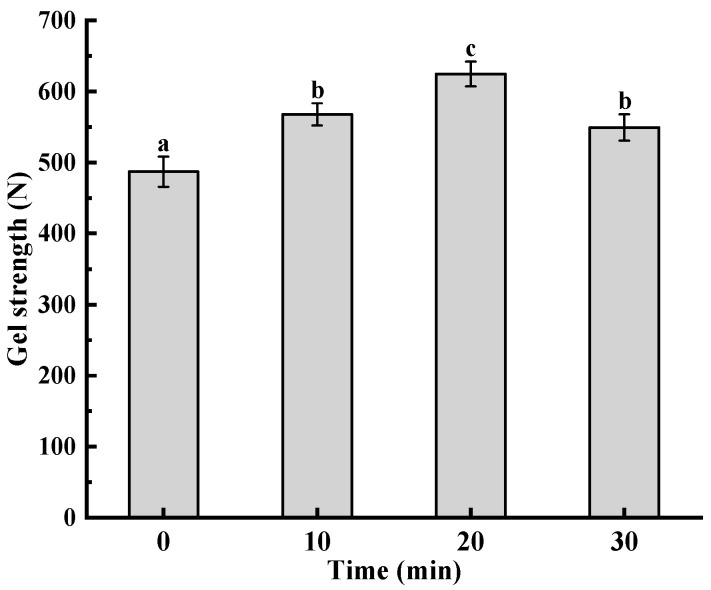
Effect of ultrasonic treatment time on the gel strength of pea starch. Different letters above the bar indicate a significant difference (*p* < 0.05).

Ultrasonic treatment has an obvious effect on the texture properties of starch gel. Song et al. found that the gel strength of Pickering emulsion gels developed by sorghum flour was proportional to the amount of sorghum flour added [27]. In this study, the starch gel strength increased with the extension of ultrasonic time (Figure 6). This could be attributed to the fact that the short-chain molecules produced under the action of ultrasound easily interacted with each other, which enhanced the strength of the starch gel. However, when the ultrasonic time was 30 min, the gel strength of the starch began to decrease due to the excessive degradation of the starch molecular chain.

## 4. Conclusions

Ultrasonic treatment had significant effects on the structure and physicochemical properties of pea starch. Due to the hydrolysis caused via ultrasound, the surfaces of the starch particles were sunken, meaning they could easily absorb water, expand and aggregate, resulting in an increase in particle size. With the extension of treatment time, the crystallinity of starch slightly decreased, and the peak viscosity, trough viscosity, cold viscosity, breakdown viscosity and setback viscosity declined significantly, but the pasting temperature remained stable. And, suitable ultrasonic treatment could improve the strength of starch gel.

## Figures and Tables

**Table 1:** Effect of ultrasonic treatment time on the size distribution of pea starch.

Time	D_10_ (μm)	D_50_ (μm)	D_90_ (μm)	D_4,3_ (μm)	D_3,2_ (μm)
0 min	11.300 ± 0.026 ^b^	18.517 ± 0.032 ^c^	25.517 ± 0.015 ^c^	18.233 ± 0.021 ^c^	14.843 ± 0.006 ^c^
10 min	11.447 ± 0.025 ^b^	18.697 ± 0.025 ^c^	25.640 ± 0.030 ^c^	18.387 ± 0.032 ^c^	14.943 ± 0.021 ^c^
20 min	12.107 ± 0.021 ^a^	19.123 ± 0.015 ^b^	25.837 ± 0.015 ^b^	18.837 ± 0.015 ^b^	15.693 ± 0.025 ^b^
30 min	12.293 ± 0.021 ^a^	20.900 ± 0.020 ^a^	29.857 ± 0.015 ^a^	20.853 ± 0.025 ^a^	16.627 ± 0.025 ^a^

D_10_, D_50_ and D_90_ mean that 10%, 50% and 90% of the particle sizes are below the measured size values, respectively. D_4,3_ and D_3,2_ are volume mean diameter and area mean diameter, respectively. Different letters in the same column indicate a significant difference (*p* < 0.05).

**Table 2:** Pasting characteristics of pea starch at different ultrasound times.

	0	10 min	20 min	30 min
Pasting temperature (°C)	75.33 ± 0.03 ^a^	75.30 ± 0.00 ^a^	75.28 ± 0.03 ^a^	75.32 ± 0.03 ^a^
Peak viscosity (cp)	3990 ± 32.58 ^a^	3756 ± 20.13 ^b^	3433 ± 32.79 ^d^	3582 ± 16.04 ^c^
Trough viscosity (cp)	3012 ± 10.00 ^a^	2818 ± 18.72 ^b^	2690 ± 11.68 ^c^	3070 ± 27.62 ^a^
Cold viscosity (cp)	6052 ± 47.60 ^a^	5550 ± 42.44 ^b^	4819 ± 33.26 ^c^	5596 ± 8.54 ^b^
Breakdown viscosity (cp)	978 ± 23.44 ^a^	919 ± 17.01 ^b^	740 ± 23.35 ^c^	491 ± 6.43 ^d^
Setback viscosity (cp)	3075 ± 60.10 ^a^	2722 ± 31.43 ^b^	2127 ± 25.17 ^d^	2484 ± 20.11 ^c^

Different letters in the same row indicate a significant difference (*p* < 0.05).

## Data Availability

Data is contained within the article.
